# Comparative genomics analysis of pheophorbide *a* oxygenase (*PAO*) genes in eight pyrus genomes and their regulatory role in multiple stress responses in Chinese pear (*Pyrus bretschneideri*)

**DOI:** 10.3389/fgene.2024.1396744

**Published:** 2024-04-16

**Authors:** Yuchen Ma, Jiao Sun, Xiao Zhang, Muhammad Sadaqat, Muhammad Tahir Ul Qamar, Tingting Liu

**Affiliations:** ^1^ College of Horticulture, Shanxi Agricultural University, Jinzhong, China; ^2^ Engineering Research Center of Coal-Based Ecological Carbon Sequestration Technology of the Ministry of Education, Shanxi Datong University, Datong, China; ^3^ Key Laboratory of Graphene Forestry Application of National Forest and Grass Administration, Shanxi Datong University, Datong, China; ^4^ Integrative Omics and Molecular Modeling Laboratory, Department of Bioinformatics and Biotechnology, Government College University Faisalabad (GCUF), Faisalabad, Pakistan

**Keywords:** pyrus, chlorophyll breakdown, pangenome-wide, PAO, fruit hardening, abiotic stress, drought stress

## Abstract

Pyrus (pear) is among the most nutritious fruits and contains fibers that have great health benefits to humans. It is mostly cultivated in temperate regions globally and is highly subjected to biotic and abiotic stresses which affect its yield. Pheophorbide a oxygenase (PAO) is an essential component of the chlorophyll degradation system and contributes to the senescence of leaves. It is responsible for opening the pheophorbide a porphyrin macrocycle and forming the main fluorescent chlorophyll catabolite However, this gene family and its members have not been explored in Pyrus genomes. Here we report a pangenome-wide investigation has been conducted on eight Pyrus genomes: Cuiguan, Shanxi Duli, Zhongai 1, Nijisseiki, Yunhong No.1, d’Anjou, Bartlett v2.0, and Dangshansuli v.1.1. The phylogenetic history, their gene structure, conservation patterns of motifs, their distribution on chromosomes, and gene duplication are studied in detail which shows the intraspecific structural conservation as well as evolutionary patterns of Pyrus PAOs. *Cis*-elements, protein–protein interactions (PPI), and the Gene Ontology (GO) enrichment analyses show their potential biological functions. Furthermore, their expression in various tissues, fruit hardening conditions, and drought stress conditions is also studied. Based on phylogenetics, the identified PAOs were divided into four groups. The expansion of this gene family in Pyrus is caused by both tandem and segmental duplication. Moreover, positive and negative selection pressure equally directed the gene’s duplication process. The Pyrus *PAO* genes were enriched in hormones-related, light, development, and stress-related elements. RNA-seq data analysis showed that *PAOs* have varied levels of expression under diseased and abiotic stress conditions. The 3D structures of PAOs are also predicted to get more insights into functional conservation. Our research can be used further to get a deeper knowledge of the PAO gene family in Pyrus and to guide future research on improving the genetic composition of Pyrus to enhance stress tolerance.

## 1 Introduction

Chlorophyll breakdown has been a very old enigma, and chlorophyll disappears without any noticeable traces (Hörtensteiner and Kräutler, 2011). This phenomenon is crucial catabolic process required for fruit ripening and leaf senescence. The recent identification of a chlorophyll breakdown mechanism that is highly conserved in land plants was made possible by the structural elucidation of colorless linear tetrapyrroles as final breakdown products of chlorophyll. The primary enzyme that opens the chlorin macrocycle of pheophorbide, giving all subsequent breakdown products their characteristic, is PAO. The necessity for a senescing cell to detoxify the potentially harmful pigment was used to explain the degradation of chlorophyll; however, new studies in leaves and fruits suggest that chlorophyll catabolites may have physiological functions (Jiao et al., 2020).

Degradation of chlorophyll causes a loss of green hue, which is the most obvious sign of leaf senescence. It has been believed that PAO is an essential enzyme in the breakdown of chlorophyll (Hörtensteiner, 2006). It ultimately creates the principal fluorescent chlorophyll catabolite (FCC) by oxygenolytically cleaving the porphyrin macrocycle of pheophorbide (pheide) (Xiao et al., 2015). PAO genes were first identified in maize (*ZmLls1*) (P. Li et al., 2006) followed by other species including rice (Tang et al., 2011), tomato (Spassieva and Hille, 2002), canola (Ho et al., 2006), wheat (Ma et al., 2012), and soybean (P. Li et al., 2006). Previous research demonstrated that environmental stressors and natural senescence in plants cause the production of PAO (Pruzinská et al., 2005). Lethal leaf spot 1 (*LLS1*) of maize and *AtPAO*, which is represented by the accelerated cell death 1 (*ACD1*) gene in Arabidopsis, are similar. It is a member of the tiny family of iron-sulfur oxygenases of the Rieske type (Pruzinská et al., 2003). Senescence caused in persistent darkness has been demonstrated to accumulate PAO and cause light-independent cell death in the absence of *ACD1* (Tanaka et al., 2003; Hirashima et al., 2009). Recent researches on *PAO* have focused on the functional studies to inhibit the cell death. The Papper *PAO* gene express itself in response to different stressors and natural senescence. The *CaPAO* gene may be crucial in salt-induced leaf senescence and defense responses to a variety of stressors (Xiao et al., 2015).

The porphyrin macrocycle is opened by PaO, a nonheme iron monooxygenase found in the inner envelope of developing gerontoplasts, by introducing two oxygen atoms. Measurements of PaO activity have demonstrated that Pheide *a* is an effective substrate, while Pheide *b* functions as a competitive inhibitor (Ho et al., 2006). *AtPaO* is a five-member gene family in Arabidopsis that codes for nonheme iron oxygenases, which are distinguished by the coexistence of a mononuclear iron-binding domain and a Rieske-type domain. Additionally, this gene family contains Tic55, PTC52, choline monoxygenase, and chloroacetate oxygenase. This gene family also contains choline monooxygenase, Chl a oxygenase, Ptc52, and Tic55 (Gray et al., 2004).

Pears stand one of the most significant temperate fruit trees globally and belong to the Rosaceae family and the *Amygdaloideae* subfamily. Pears have been cultivated for over 3,000 years with 39 billion tons are delievered worldwide every year (Wu et al., 2014; 2018; Ferradini et al., 2017). Presently, 22 species of pear with 5000 accessions have been reported. Among these five species are majorly cultivated for fruit production, including *Pyrus* bretschneideri, *P*. *pyrifolia*, *P. communis*, *P. ussuriensis* and *P. sinkiangensis* (Li et al., 2022). The majority of cultivated pears have a diploid genome (2n = 34), which is extremely heterozygous and has several repeating sequences (S. Chen et al., 2023). In this project, eight pear genomes have been used; Cuiguan (*P. pyrifolia*), Shanxi Duli (*P. betulifolia*), Zhongai 1 [(*P. ussuriensis* × *communis*) × spp.], Nijisseiki (*P. pyrifolia*), Yunhong No.1 (*P. pyrifolia*), d’Anjou (*P. communis*), Bartlett v2.0 (*P. communis*), Dangshansuli’ v.1.1 (*P. bretschneideri*) have been used (S. Chen et al., 2023). Pear is an economical fruit with sweet taste and great nutritional value with a cultivation history of up to 3000 years back. However, no research is available on Pyrus *PAO* genes and their regulatory mechanism. Thus, this study provides information regarding the characterization of PAO gene family members from multiple Pyrus genomes, to understand their evolution, intra-specific, and functional diversity. Further, the expression profiles of Pyrus *PAO* genes in tissues, abiotic, and biotic stresses have been determined, providing valuable insights for future stress-resistant pear breeding. This comprehensive study will be useful for further functional investigations of Pyrus *PAOs*.

## 2 Materials and methods

### 2.1 Identification of PAO genes

The protein sequences of *Arabidopsis thaliana* PAO proteins were obtained from The TAIR database (https://www.arabidopsis.org/). The protein sequence FASTA files of eight Pyrus species including Cuiguan, Shanxi Duli, Zhongai 1, Nijisseiki, Yunhong No.1, d’Anjou, Bartlett v2.0, Dangshansuli’ v.1.1 were used as subject sequences to run the command-line tool, BLAST+. A BLASTp search was conducted against the eight Pyrus proteomes using AtPAO protein sequences as the queries. The hits from this search were then refined, including the removal of isoforms and duplicates.

The Pyrus PAO candidate sequences were examined for domains through the NCBI Conserved Domain Database (CDD; https://www.ncbi.nlm.nih.gov/Structure/cdd/cdd.shtml) (Marchler-Bauer et al., 2011) and InterPro (https://www.ebi.ac.uk/interpro/) (Hunter et al., 2009) to identify the definitive protein family sequences. Physicochemical properties such as molecular weight (MW), isoelectric point (pI), aliphatic index (AI), instability index (II), and the grand average of hydropathicity (GRAVY) were forecasted using the ExPASy ProtParam tool (https://web.expasy.org/protparam/) (Gasteiger et al., 2005). The subcellular localization of these PAO proteins was forecasted using the WoLF PSORT tool (https://wolfpsort.hgc.jp/) (Horton et al., 2007).

### 2.2 Multiple sequence alignment, phylogenetic analysis, gene structure and conserved motifs analysis

Phylogenetic analysis was carried out to assess the intra-specific evolutionary connections among Pyrus PAOs. A multiple sequence alignment of 11 Cuiguan, 8 Shanxi Duli, 10 Zhongai 1, 10 Nijisseiki, 10 Yunhong No.1, 9 d'Anjou, 5 Bartlett v2.0, 8 Dangshansuli’ v.1.1, 4 *A. thaliana*, 5 *O. sativa*, and 4 *Z. mays* protein sequences was performed using ClustalW (Thompson et al., 2003). The phylogenetic tree was constructed using the IQTREE Web Server (http://iqtree.cibiv.univie.ac.at/) (Trifinopoulos et al., 2016) with the maximum likelihood (ML) method and bootstrap replicates of 1000. The iTOL: Interactive Tree of Life (https://itol.embl.de/) (Letunic and Bork, 2007) was used for editing and visualization.

To identify the conserved common motifs in all eight Pyrus PAO sequences, the MEME tool (https://meme-suite.org/meme/) (Bailey et al., 2009) was utilized. The number of conserved motifs was set to 20 for each sequence. Gene structures were constructed using CDS and genomic sequences through the Gene Structure Display Server (GSDS; https://gsds.gao-lab.org/) (Hu et al., 2015). The identified motifs and gene structures were visualized using TBtools-II v2.067 (C. Chen et al., 2018).

### 2.3 Chromosomal mapping and duplication events analysis

The chromosomal positions of each Pyrus *PAOs* were extracted from GFF/GFF3 files and mapped to chromosomes using TBtools-II v2.067: Gene Location Visualize advanced (C. Chen et al., 2018). By assessing whether the shorter gene’s length covered 70% of the longer gene and if the alignment similarity between the two genes was 70% or higher, instances of *PAOs* gene duplication were identified (ul Qamar et al., 2023). The duplication pattern, whether segmental or tandem, was also examined. To predict the selection pressure on the duplicated genes, Ka/Ks values were calculated using DnaSP v.6 software (Le et al., 2011; Rozas et al., 2017). Based on whether the Ka/Ks ratio was greater than, equal to, or less than one, purifying, neutral, or positive selection was inferred (Zia et al., 2022). Additionally, the divergence time for the duplicated gene pairs was estimated using the formula “t = Ks/2λ×10^–6^″ in million years (Mya), where the λ value is 1.5 × 10^−8^ for dicots (Fatima et al., 2023; Sadaqat et al., 2023).

### 2.4 PPI and GO enrichment analysis

The amino acid sequences of the Pyrus PAOs proteins were analyzed for protein-protein interactions (PPIs) using the STRING database (Mering et al., 2003). The top ten interactions were selected for prediction, with an interaction threshold set at 0.4. The PPI network was then visualized using Cytoscape software (Shannon et al., 2003). Additionally, the PANNZER database (http://ekhidna2.biocenter.helsinki.fi/sanspanz/) (Törönen et al., 2018) was utilized to predict the GO enrichment analysis components, including biological processes (BPs), cellular components (CCs), and molecular functions (MFs).

### 2.5 *Cis*-regulatory elements prediction and expression analysis of pyrus *PAOs*

To predict the *cis*-regulatory elements, the 2 kb sequences upstream of the translation start site of Pyrus PAOs genes were obtained and analyzed using the PlantCARE database (https://bioinformatics.psb.ugent.be/webtools/plantcare/html/) (Rombauts et al., 1999).

To understand the expression patterns of Pyrus Dangshansuli PAOs, transcriptomic RNA-seq data from different developmental stages of pear fruit (BioProject: PRJNA309745), under drought stress (BioProject: PRJNA655255), and Fruit hardening disease (BioProject: PRJNA763913) were retrieved from the SRA-NCBI database (https://www.ncbi.nlm.nih.gov/sra). The genome and annotation (GFF) files of Dangshansuli were downloaded from “The pear genomics database” (PGDB; http://pyrusgdb.sdau.edu.cn/) (S. Chen et al., 2023). The quality of reads was assessed using the FastQC tool (Wingett and Andrews, 2018). HISAT2 (Kim et al., 2019) was used to build the indexes of the Pyrus genome, and the high-quality paired-clean reads were then mapped onto the indexed genome. The abundance estimation of gene family members was carried out using StringTie (Pertea et al., 2016). Finally, a heatmap was generated using the Fragments Per Kilobase of transcript per Million mapped reads (FPKM) values (Zameer et al., 2021).

### 2.6 3D structure prediction of DaPAO proteins

To ensure proper functionality, proteins require a three-dimensional (3D) structure. The 3D structures of eight Dangshansuli PAOs (DaPAO1-DaPAO8) were predicted using AlphaFold2 (https://alphafold.ebi.ac.uk/) (Jumper et al., 2021). The accuracy of these predicted structures was assessed through the SAVES server (https://saves.mbi.ucla.edu/) (Zameer et al., 2022) and MolProbity (http://molprobity.biochem.duke.edu/) (Davis et al., 2007). Finally, the UCSF ChimeraX software (Goddard et al., 2018) was utilized to visualize these structures (Neupane et al., 2018; Zia et al., 2024).

## 3 Results

### 3.1 Identification of PAO genes in eight pyrus species

A total of 11 genes from the Cuiguan genome (CuPAO), eight from Shanxi Duli (ShPAO), 10 from Zhongai1 (ZhPAO), 10 from Nijisseiki (NiPAO), 10 from Yunhong No.1 (YuPAO), nine from d’Anjou (AnPAO), five from Bartlett v2.0 (BrPAO), and eight from Dangshansuli’ v.1.1 genome (DaPAO) were identified. All of these members were confirmed to have the Rieske [2Fe-2S] iron-sulphur domain superfamily and Pheophorbide a oxygenase (PAO) domain. The protein names of each member were assigned based on their position on chromosomes (Table 1).

**TABLE 1:** The PAO gene family members identified in eight Pyrus species, their physicochemical characteristics, and subcellular localization.

Name	Gene	Chr	Start	End	Strand	AA	MW (kDa)	pI	II	AI	GRAVY	Subcellular Location
**Cuiguan**												
CuPAO1	EVM0015613	Chr3	29306982	29310103	-	538	60.30	8.54	45.16	83.72	−0.198	Plasma membrane
CuPAO2	EVM0034374	Chr3	29699416	29702567	+	521	58.44	8.46	45.23	83.26	−0.220	Plasma membrane
CuPAO3	EVM0003339	Chr8	7814776	7817938	+	542	61.10	8.44	45.33	78.62	−0.362	Chloroplast
CuPAO4	EVM0021838	Chr8	21839328	21841494	+	405	46.28	8.12	44.21	73.14	−0.638	Nucleus
CuPAO5	EVM0001085	Chr8	21846296	21849078	+	557	62.52	8.77	41.17	78.99	−0.330	Plasma membrane
CuPAO6	EVM0000633	Chr11	417414	421294	-	537	60.41	8.89	43.27	79.57	−0.254	Plasma membrane
CuPAO7	EVM0013357	Chr11	421653	425314	-	536	60.38	8.84	37.27	76.79	−0.289	Chloroplast
CuPAO8	EVM0030664	Chr11	10620931	10626688	-	545	61.17	5.89	47.58	69.82	−0.419	Chloroplast
CuPAO9	EVM0011128	Chr13	15175223	15177961	-	342	38.96	6.23	35.32	76.64	−0.335	Mitochondria
CuPAO10	EVM0001870	Chr15	9622350	9625444	+	493	56.30	9.05	47.46	73.55	−0.455	Chloroplast
CuPAO11	EVM0039391	Chr15	42227819	42229991	+	575	64.14	8.96	47.46	74.16	−0.369	Vacuole membrane
**Shanxi Duli**												
ShPAO1	Chr3.g20819	Chr3	369968	376891	-	1202	134.87	8.28	49.73	78.92	−0.376	Plasma membrane
ShPAO2	Chr8.g54046	Chr8	3293817	3295116	+	281	32.06	9.34	45.63	77.65	−0.528	Nucleus
ShPAO3	Chr8.g54047	Chr8	3308513	3311530	+	573	64.09	8.77	42.79	78.32	−0.327	Chloroplast
ShPAO4	Chr11.g09723	Chr11	482356	490252	-	536	60.40	8.84	38.07	77.33	−0.282	Chloroplast
ShPAO5	Chr11.g11184	Chr11	13495121	13500826	-	545	61.22	5.99	47.23	69.82	−0.432	Chloroplast
ShPAO6	Chr13.g22184	Chr13	18348814	18351742	+	405	45.88	5.96	36.93	74.59	−0.309	Mitochondria
ShPAO7	Chr15.g04534	Chr15	2577622	2579735	-	555	62.22	8.92	43.23	75.08	−0.382	Plasma membrane
ShPAO8	Chr15.g01258	Chr15	33455819	33459232	-	416	46.94	6.60	47.42	79.88	−0.411	Nucleus
**Zhongai 1**												
ZhPAO1	Pdr3g017730	Chr3	21297828	21302446	-	781	87.75	8.26	55.71	72.29	−0.491	Nucleus
ZhPAO2	Pdr8g003030	Chr8	2410247	2413029	-	557	62.53	8.77	40.48	78.99	−0.329	Chloroplast
ZhPAO3	Pdr8g003040	Chr8	2417842	2419575	-	369	42.42	6.95	41.60	82.11	−0.467	Cytoplasm
ZhPAO4	Pdr8g014970	Chr8	15254617	15257779	+	542	61.02	8.55	44.85	78.43	−0.367	Chloroplast
ZhPAO5	Pdr11g016070	Chr11	20715645	20718149	+	190	20.98	4.76	53.76	70.95	−0.299	Chloroplast
ZhPAO6	Pdr11g016080	Chr11	20725281	20730911	+	406	45.83	7.11	41.48	70.62	−0.361	Chloroplast
ZhPAO7	Pdr11g025050	Chr11	29454346	29458047	+	536	60.52	8.84	38.80	79.51	−0.248	Chloroplast
ZhPAO8	Pdr11g025060	Chr11	29458494	29462014	+	367	41.42	8.33	44.76	80.76	−0.228	cytoskeleton
ZhPAO9	Pdr13g009780	Chr13	9835670	9838575	+	405	45.99	6.07	35.85	74.35	−0.325	Mitochondria
ZhPAO10	Pdr0g034120	contig15	3260	5434	-	575	64.20	8.89	47.25	73.48	−0.377	Vacuole membrane
**Nijisseiki**												
NiPAO1	Ppy03g0046.1	Chr3	466995	470149	-	528	59.24	8.63	47.11	82.54	−0.190	Plasma membrane
NiPAO2	Ppy08g0407.1	Chr8	2892290	2893339	+	174	19.99	5.65	45.31	82.30	−0.601	Cytoplasm
NiPAO3	Ppy08g0409.1	Chr8	2898825	2901502	+	573	64.13	8.77	42.79	77.64	−0.328	Chloroplast
NiPAO4	Ppy08g1602.1	Chr8	15926551	15929714	-	542	61.10	8.44	45.33	78.62	−0.362	Chloroplast
NiPAO5	Ppy11g0057.1	Chr11	419458	427174	-	993	111.40	8.86	38.76	76.33	−0.271	Vacuole membrane
NiPAO6	Ppy11g1156.1	Chr11	10869281	10874503	-	545	61.14	5.89	47.58	70.53	−0.416	Chloroplast
NiPAO7	Ppy11g1157.1	Chr11	10880121	10882280	-	186	21.18	4.77	55.04	64.03	−0.666	Chloroplast
NiPAO8	Ppy13g2286.1	Chr13	19204973	19207914	+	406	45.96	6.02	34.62	74.16	−0.314	Mitochondria
NiPAO9	Ppy15g0353.1	Chr15	2351374	2353546	-	575	64.14	8.96	47.46	74.16	−0.369	Vacuole membrane
NiPAO10	Ppy15g2909.1	Chr15	30146765	30149606	-	514	58.65	6.99	44.51	76.60	−0.457	Cytoplasm
**Yunhong No. 1**												
YuPAO1	Pspp.Chr03.00051	Chr3	462727	470681	-	1212	135.69	8.35	49.69	78.83	−0.367	Plasma membrane
YuPAO2	Pspp.Chr08.00369	Chr8	3150296	3152027	+	331	38.11	8.74	40.88	79.18	−0.476	Endoplasmic reticulum
YuPAO3	Pspp.Chr08.00370	Chr8	3156972	3159606	+	557	62.47	8.77	40.53	79.35	−0.332	Chloroplast
YuPAO4	Pspp.Chr08.01302	Chr8	16302930	16306086	-	542	61.05	8.73	46.08	78.25	−0.373	Chloroplast
YuPAO5	Pspp.Chr11.00058	Chr11	424117	432146	-	537	60.45	9.03	40.68	77.75	−0.275	Chloroplast
YuPAO6	Pspp.Chr11.00979	Chr11	10746610	10751832	-	545	61.14	5.89	47.58	70.53	−0.416	Chloroplast
YuPAO7	Pspp.Chr11.00981	Chr11	10757440	10761088	-	268	30.21	4.69	42.07	68.10	−0.389	Chloroplast
YuPAO8	Pspp.Chr13.01994	Chr13	17520787	17523714	+	405	45.96	6.07	35.80	74.59	−0.332	Mitochondria
YuPAO9	Pspp.Chr15.00363	Chr15	2497717	2499994	-	555	62.32	8.82	44.19	75.77	−0.363	Plasma membrane
YuPAO10	Pspp.Chr15.02995	Chr15	31963634	31967135	-	436	49.50	7.63	44.25	78.90	−0.436	Nucleus
**d’Anjou**												
AnPAO1	DAnjou_Chr3v0.1_05362	Chr3	501982	513103	-	1628	184.99	8.20	45.57	77.12	−0.454	Plasma membrane
AnPAO2	DAnjou_Chr8v0.1_19235	Chr8	3877524	3879035	+	240	27.76	6.40	40.12	73.46	−0.749	Chloroplast
AnPAO3	DAnjou_Chr8v0.1_20292	Chr8	16115524	16119085	-	542	60.96	8.55	45.64	78.62	−0.369	Chloroplast
AnPAO4	DAnjou_Chr8v0.1_20299	Chr8	16231075	16234647	-	542	61.00	8.73	45.73	78.62	−0.363	Chloroplast
AnPAO5	DAnjou_Chr11v0.1_27769	Chr11	15878132	15884101	-	545	61.21	5.89	45.67	70.53	−0.412	Chloroplast
AnPAO6	DAnjou_Chr11v0.1_27770	Chr11	15892188	15894292	-	275	30.75	5.45	52.26	75.24	−0.204	Plasma membrane
AnPAO7	DAnjou_Chr13v0.1_33805	Chr13	20463579	20466625	+	405	45.88	6.07	36.83	74.59	−0.322	Chloroplast
AnPAO8	DAnjou_Chr15v0.1_36607	Chr15	2602795	2604955	-	539	60.13	8.86	48.16	72.43	−0.381	Chloroplast
AnPAO9	DAnjou_Chr15v0.1_39375	Chr15	35062568	35066303	-	576	65.13	8.20	51.90	82.95	−0.370	Nucleus
**Bartlett**												
BaPAO1	pycom08g14010	Chr8	13039687	13042245	-	419	46.94	9.07	44.79	77.06	−0.366	Chloroplast
BaPAO2	pycom11g00550	Chr11	401313	409036	-	537	60.56	9.03	40.98	78.47	−0.275	Chloroplast
BaPAO3	pycom11g12210	Chr11	11105811	11111270	-	545	61.40	6.10	47.00	70.72	−0.417	Chloroplast
BaPAO4	pycom13g21200	Chr13	17506709	17509649	+	405	45.88	5.96	36.95	74.59	−0.313	Mitochondria
BaPAO5	pycom15g31160	Chr15	29487570	29490736	-	542	61.21	8.46	44.55	78.06	−0.394	Chloroplast
**Dangshansuli**												
DaPAO1	LOC103964560	Chr3	19270268	19273854	+	538	60.30	8.54	45.16	83.72	−0.198	Plasma membrane
DaPAO2	LOC103940306	Chr8	8832874	8836530	-	542	61.03	8.44	45.47	79.15	−0.357	Chloroplast
DaPAO3	LOC103949697	Chr8	15728977	15731942	-	573	64.10	8.77	43.45	77.31	−0.333	Chloroplast
DaPAO4	LOC103954047	Chr11	4944084	4948125	+	536	60.33	8.91	38.96	77.16	−0.283	Chloroplast
DaPAO5	LOC103954046	Chr11	4948285	4952127	+	537	60.40	8.92	43.83	79.03	−0.264	Plasma membrane
DaPAO6	LOC103947727	Chr11	11153637	11159120	+	545	61.12	6.04	46.56	70.00	−0.409	Chloroplast
DaPAO7	LOC103935767	Chr13	11807237	11810606	-	406	46.04	6.07	35.95	74.66	−0.327	Mitochondria
DaPAO8	LOC103953347	Chr15	2957979	2960414	-	575	64.20	8.89	47.25	73.48	−0.377	Vacuole membrane

Bold represents the species name.

The physical and chemical properties of all Pyrus PAO proteins were computed and compared. The protein length ranged from 174–1628 aa and molecular weight ranged from 19.99 to 18.50 kDa. The isoelectric point showed that most of the proteins are basic in nature. The instability index denoted that most of these proteins are unstable. Aliphatic index showed that proteins are good thermostable. GRAVY values indicates that the proteins have hydrophilic behaviour. Subcellular localization analysis revealed that most proteins were found in the chloroplast, with a few in the nucleus, plasma membrane, mitochondria (Table 1).

### 3.2 Phylogenetic relationships of pyrus PAO family members

**FIGURE 1:**
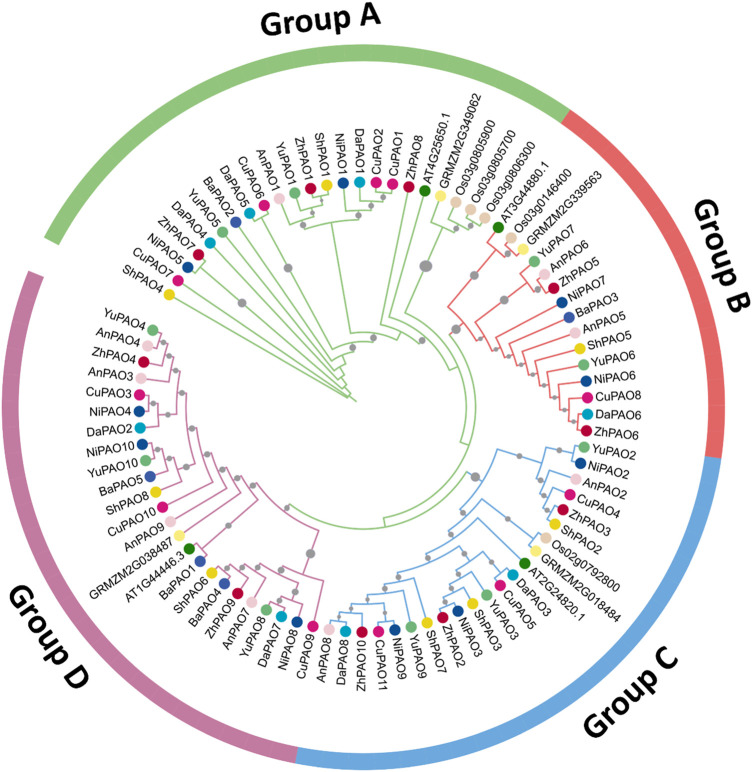
Phylogenetic tree of PAO gene family members from eight Pyrus, *A. thaliana*, *O. sativa*, and *Z. mays* genomes. Each group is represented by a particular color with specific symbol used for each species.

To examine the evolutionary connections among the PAO members from eight Pyrus genomes, a phylogenetic tree was created using 84 amino acid sequences from 11 species. All PAO proteins were grouped into four sub-family clusters: Group A, B, C, and D. The Group D cluster was the largest, with 24 members. After that the Group A was the second largest with 23 members. Group C and B has the members 22 and 15 respectively (Figure 1).

### 3.3 Gene structure and conserved motifs analysis of pyrus PAOs

**FIGURE 2:**
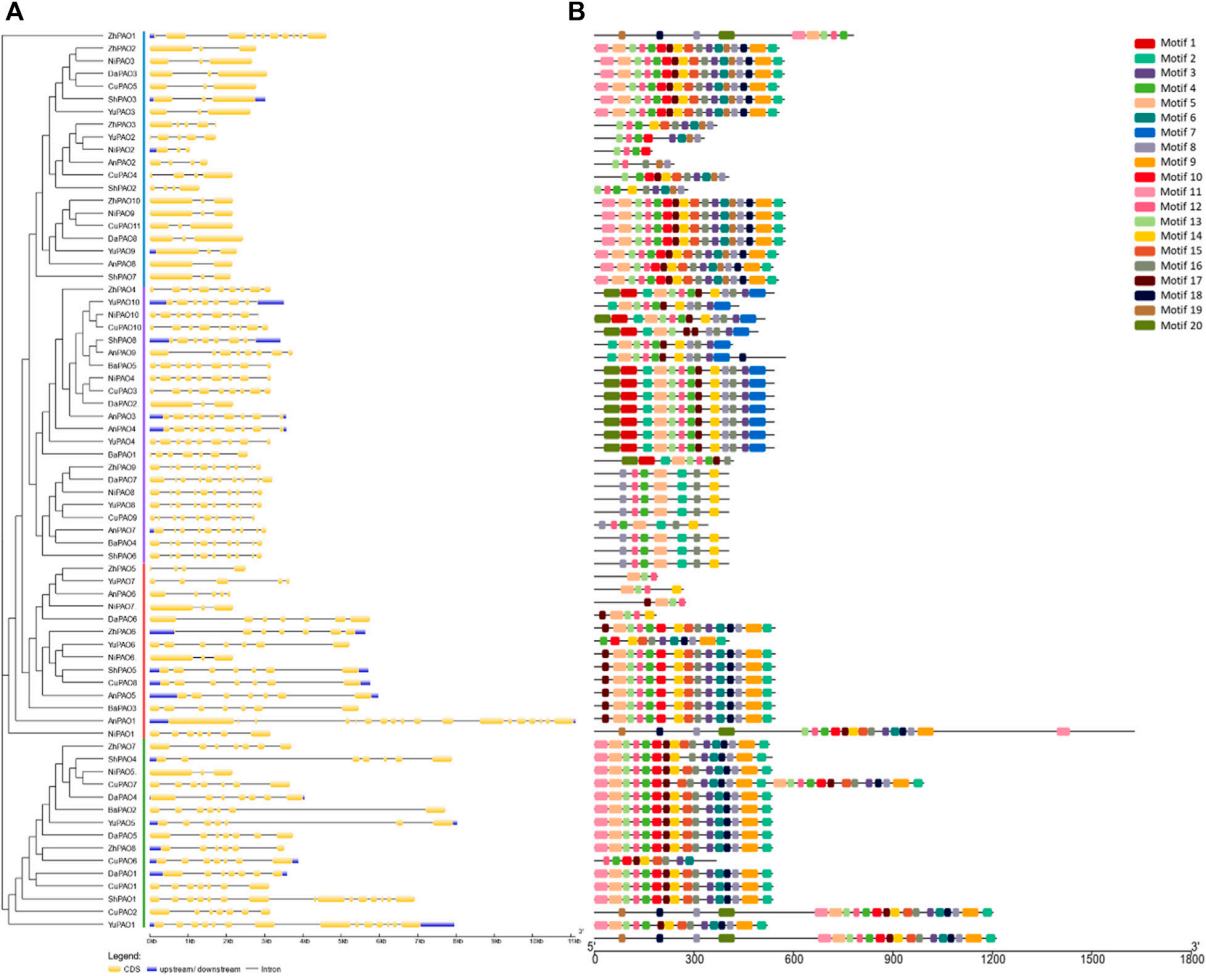
**(A)** Gene structure showing conservation pattern of exons and introns and **(B)** Pattern of conserved motifs.

To understand the evolutionary patterns among Pyrus PAOs, their conserved motifs and gene structures were studied. The gene structure was observed to be highly conserved within members of the same subfamily. The number of exons ranged from 3–18. Group A and B has more numbers of exon as compared to Group C and D (Figure 2A)**.**

All members of each subfamily shared highly conserved motifs. Members of Group A, B, C, and D had exactly the same motif pattern (conserved motifs 2, 3, 4, 5, 8, 11, 12, 14, 16). Motif seven was only present in Group D and the motifs 6, 9, 10, 13, 15, 17, and 18 were present in all members except few members of Group D (Figure 2B). This high conservation of motifs suggests no major differences in the structure and functions of Pyrus PAOs.

### 3.4 Chromosomal location and gene duplication analysis

**FIGURE 3:**
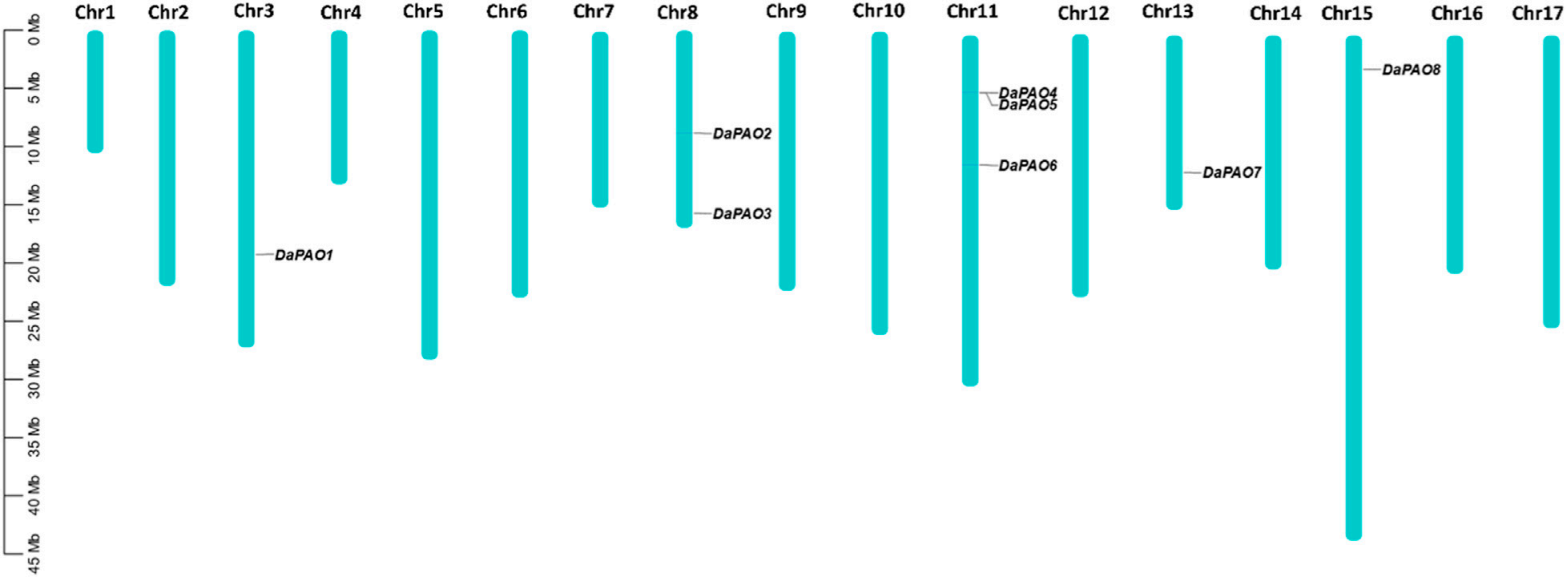
Chromosomal map showing eight *DaPAOs* distributed on five Dangshansuli’ v.1.1 chromosomes.

To assess the gene distribution pattern of Pyrus *PAOs* across the 17 chromosomes of each Pyrus genome, their chromosomal gene localization was determined. This analysis revealed an uneven distribution of genes across chromosomes. In the Cuiguan genome, 11 *CuPAOs* were localized on five out of seventeen chromosomes (Chr3, 8, 11, 13, and 15). The remaining chromosomes did not contain any *CuPAOs* genes. Similarly, in the Shanxi Duli genome, eight *ShPAOs* were scattered across five out of seventeen chromosomes. In the Zhongai1 genome, 10 *ZhPAOs* were distributed across four out of seventeen chromosomes. In the Nijisseiki genome, 10 *NiPAOs* were distributed across four chromosomes. In the Yunhong No.1 genome, 10 *YuPAOs* were unevenly distributed across four chromosomes. In the d’Anjou genome, nine *AnPAOs* were distributed across five chromosomes. In the Bartlett v2.0 genome, five *BaPAOs* were distributed across four chromosomes. Finally, in the Dangshansuli’ v.1.1 genome, eight *DaPAOs* members were distributed across five out of seventeen chromosomes (Figure 3).

Gene duplication events were investigated within each Pyrus *PAO* gene family member. In the Cuiguan genome, eight pairs of genes were found to be duplicated, in which two pairs duplicated through tandem duplication and six pairs duplicated through segmental duplication. The Shanxi Duli genome contained two segmentally duplicated pairs of *ShPAOs*. In the Zhongai1 genome, three pairs of *ZhPAOs* were duplicated, with two pairs being tandemly duplicated and the one pair being segmentally duplicated. The Nijisseiki genome had two pairs of segmentally duplicated *NiPAOs*. In the Yunhong No.1 genome, three pairs of *YuPAOs* were found, all originating from segmental duplication. The d'Anjou genome exhibited four pairs of duplicated genes, with two pairs being tandemly duplicated and the other two pairs segmentally duplicated. The Bartlett genome contained only one pair of segmentally duplicated genes. The Dangshansuli genome had four pairs in which three pairs are segmentally duplicated and one gene pair is tandemly duplicated (Table 2).

**TABLE 2:** Duplication data of Pyrus *PAOs*, rate of synonymous (Ka) and non-synonymous mutations (Ks), duplication time (Mya), and type of duplication.

Gene 1	Gene 2	Ka/Ks	Duplication time (Mya)	Duplication type
*CuPAO1*	*CuPAO2*	0.91	55.30	Tandem
*CuPAO1*	*CuPAO6*	0.65	4.95	Segmental
*CuPAO1*	*CuPAO7*	0.58	5.52	Segmental
*CuPAO2*	*CuPAO6*	1.05	48.24	Segmental
*CuPAO2*	*CuPAO7*	1.05	46.76	Segmental
*CuPAO3*	*CuPAO10*	0.49	4.56	Segmental
*CuPAO5*	*CuPAO11*	0.78	12.52	Segmental
*CuPAO6*	*CuPAO7*	0.59	2.08	Tandem
*ShPAO1*	*ShPAO4*	0.83	41.11	Segmental
*ShPAO3*	*ShPAO7*	0.77	64.60	Segmental
*ZhPAO2*	*ZhPAO10*	0.72	12.20	Segmental
*ZhPAO5*	*ZhPAO6*	0.62	54.32	Tandem
*ZhPAO7*	*ZhPAO8*	0.76	1.88	Tandem
*NiPAO3*	*NiPAO9*	0.51	91.51	Segmental
*NiPAO4*	*NiPAO10*	1.02	2.42	Segmental
*YuPAO1*	*YuPAO5*	0.98	3.87	Segmental
*YuPAO3*	*YuPAO9*	0.96	58.55	Segmental
*YuPAO4*	*YuPAO10*	0.87	3.73	Segmental
*AnPAO3*	*AnPAO4*	0.83	0.50	Tandem
*AnPAO3*	*AnPAO5*	0.87	54.53	Segmental
*AnPAO4*	*AnPAO9*	0.48	4.74	Segmental
*AnPAO5*	*AnPAO6*	0.55	55.69	Tandem
*BaPAO1*	*BaPAO5*	0.79	4.04	Segmental
*DaPAO1*	*DaPAO4*	0.97	4.62	Segmental
*DaPAO1*	*DaPAO5*	0.84	3.58	Segmental
*DaPAO3*	*DaPAO8*	0.90	5.65	Segmental
*DaPAO4*	*DaPAO5*	0.99	2.29	Tandem

Italic represents the gene names.

To investigate the evolutionary pressures acting on the duplicated Pyrus *PAOs* genes, the Ka, Ks, and Ka/Ks ratios were computed for all para-homologous gene pairs. Across the eight genomes, the Ka/Ks ratio varied from 0.48 to 1.05, indicating a mix of positive and negative selection events. The divergence time of the 27 duplicated gene pairs of Pyrus *PAOs* ranged from 0.5 to 91.51 million years ago (MYA) (Table 2).

### 3.5 PPI and GO enrichment analysis

**FIGURE 4:**
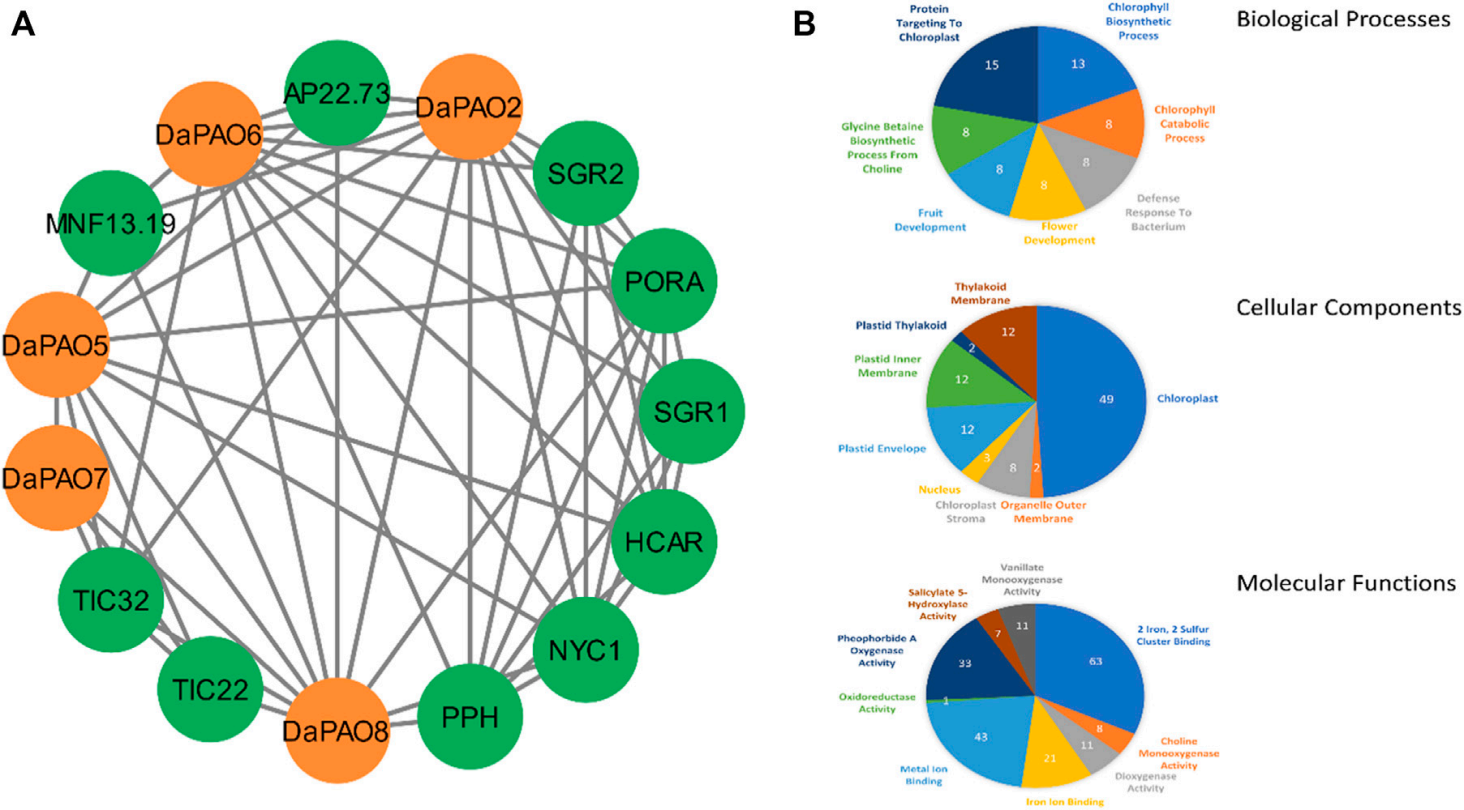
**(A)** Interactions among Pyrus PAOs and other homologous proteins. **(B)** Predicted BPs, CCs, and MFs associated with Pyrus PAOs.

A protein-protein interaction (PPI) network of the Pyrus PAOs was constructed to explore the functional diversity among its members. Among the five Dangshansuli members, DaPAO2, DaPAO5, DaPAO6, DaPAO7, and DaPAO8, interactions with several other proteins were observed. The majority of these interactions were identified with PORA (degree 9) and NYC1 (degree 9) proteins, suggesting the potential roles of these Pyrus members in skotomorphogenesis, photomorphogenesis and throughout the plant life under specific light conditions (Figure 4A).

GO enrichment analysis was conducted to determine the molecular functions of Pyrus *PAOs* in a dynamic context. This analysis classified the genes into three main categories: biological processes (BPs), cellular components (CCs), and molecular functions (MFs). The primary BPs identified included the protein targeting to chloroplast, chlorophyll biosynthetic process, and flower and fruit Development. The genes were found to be present in CCs such as the chloroplast, plastid envelope, and thylakoid membrane. The MFs associated with these genes included 2 Iron, two sulfur cluster binding, metal ion binding, and pheophorbide A oxygenase activity (Figure 4B, Supplementary Material S1).

### 3.6 *Cis*-regulatory element analysis of pyrus *PAOs*

**FIGURE 5:**
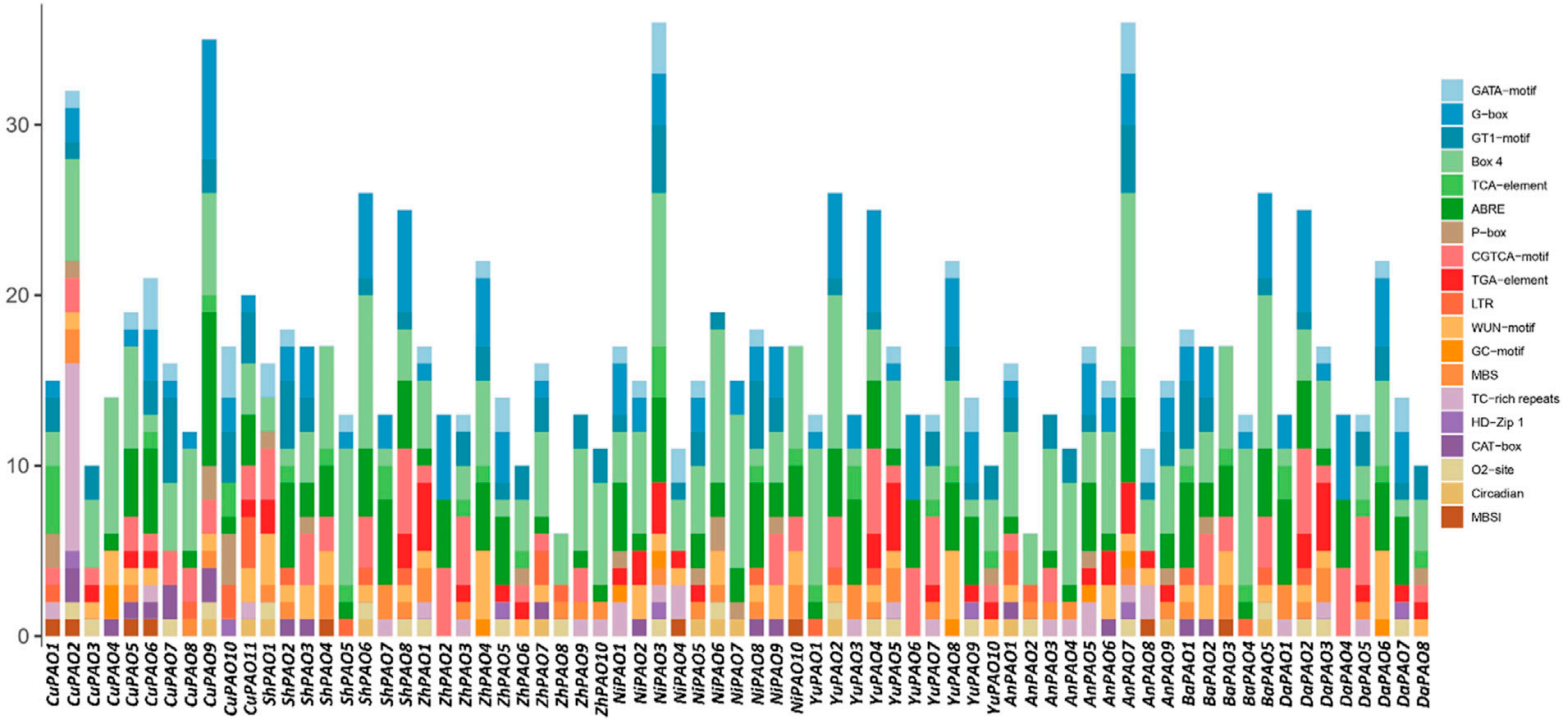
The Pyrus *PAOs* genes’ upstream promoter regions contain *cis*-regulatory elements. Each bar represents a distinct element found in a given gene.

To gain a deeper understanding of the diverse stress responses exhibited by Pyrus *PAOs*, the *cis*-regulatory elements in their promoter sequences were analyzed. Across all genomes, *cis*-elements associated with stress responses, including light, hormones, and development, were found in abundance. Specifically, G-box, GT1-motif, and GATA-motif (*c*is-element Box 4) were identified as being involved in the regulation of light-stress. Hormone responsiveness was linked to five *cis*-elements: P-box, TGA-element, ABRE, CGTCA-motif, and TCA-element. Additionally, the GC-motif, LTR, TC-rich repeats, and MBS were identified as the four *cis*-elements associated with stress responsiveness. For developmental processes, five elements were involved: CAT-box, MBSI, circadian, HD-Zip 1, and o2-site. The presence of these elements in Pyrus *PAO* suggests their involvement in hormone, stress, and development-related responses (Figure 5, Supplementary Material S2).

### 3.7 Gene expression profiling of pyrus *PAOs*

**FIGURE 6:**
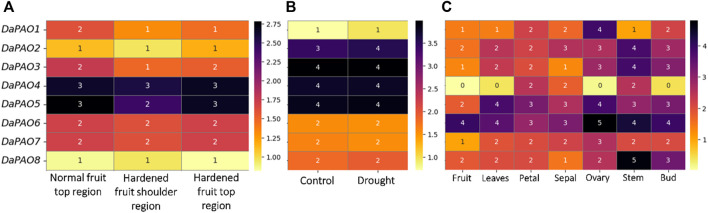
Heatmap showing the expression pattern of *DaPAOs* in **(A)** Disease condition where normal fruit region was compared with hardened fruit, **(B)** Drought stress condition, and **(C)** Different tissues, where yellow color shows downregulation and dark color shows upregulation.

Transcriptome expression data were utilized to assess the expression levels of eight *DaPAOs* under fruit hardening disease to evaluate the expression of *DaPAOs* in diseased conditions. *DaPAO4* and *DaPAO5* were highly expressed in all conditions. *DaPAO1* was downregulated in disease conditions as compared to normal condition (Figure 6A). Under drought stress, different expression levels were observed, with *DaPAO2* being upregulated. On the other hand, the DaPAOs have no change in expression during normal and drought condition (Figure 6B). Transcriptome expression data was also utilized to assess the expression levels of eight DaPAO*s* across various tissues, including fruit, leaves, petal, sepal, ovary, stem, and bud. *DaPAO1* was highly expressed in ovary. Three genes named as *DaPAO2, 3,* and *8* were highly expressed in stem. Two genes named as *DaPAO5* and *DaPAO6* were highly expressed in most of the tissues (Figure 6C). *DaPAO1*, *DaPAO3*, *DaPAO4,* and *DaPAO5* showed fluctuated and high expression patterns which make these genes potential candidates for future research.

### 3.8 3D structure prediction of DaPAO proteins

**FIGURE 7:**
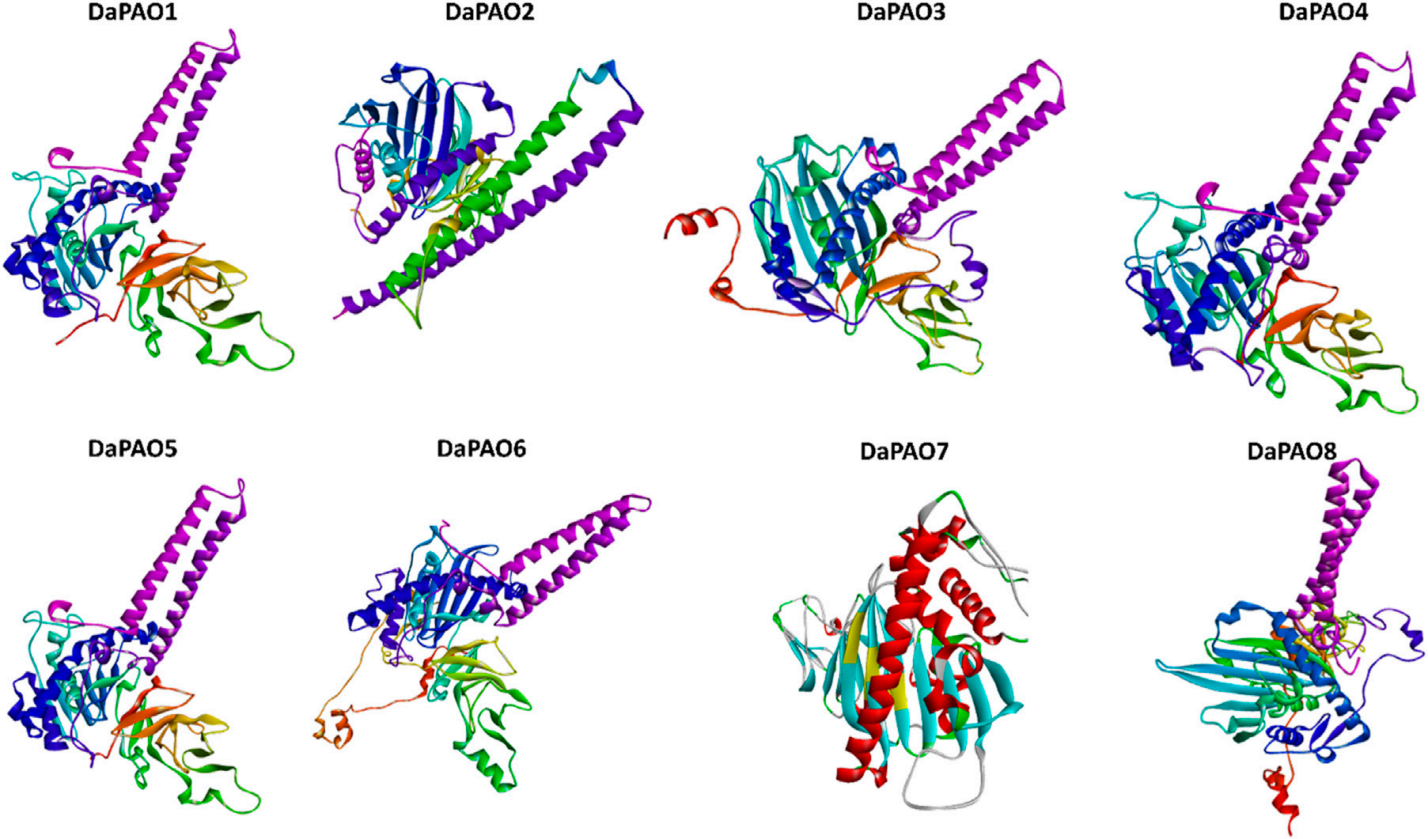
Predicted 3D structures of eight DaPAO proteins.

To delve deeper into the structural and functional diversity, the 3D structures of the eight DaPAO proteins were modeled. These proteins exhibited a high degree of conservation in their 3D structures, with similar patterns of helices and turns. Additionally, all eight proteins shared a similar helical structure at both the C and N termini. This structural conservation implies that the DaPAO proteins may have similar functions. DaPAO7 has a slightly changed structure as compared to the other DaPAOs (Figure 7).

## 4 Discussion

PAO eventually creates Fluorescent Chl catabolites (FCCs) by opening the porphyrin macrocycle of pheophorbide *a* (Hörtensteiner, 2006). In the current study, a pangenome-wide study (Tahir ul Qamar et al., 2020) of this gene family has been carried out in eight Pyrus genomes. Here we identified 11 *CuPAO* genes, 8 *ShPAO* genes, 10 *ZhPAO* genes, 10 *NiPAO* genes, 10 *YuPAO* genes, 9 *AnPAO* genes, 5 *BrPAO* genes, and 8 *DaPAO* genes. All these members contained domains conserved in all PAO homologues. One essential component of leaf senescence is the phenotypic loss of chlorophyll, and the phenotype of mutants lacking in chlorophyll breakdown makes clear how important chlorophyll degradation is. For instance, the early cell death displayed by the maize *lls1* mutant lacking in PAO eventually results in the death of the entire plant (Ougham et al., 2008; Das et al., 2018). Likewise, a phenotype of cell death is observed in rice PAO knockdown lines (Tang et al., 2011). Although the PAO/phyllobilin pathway of chlorophyll breakdown is important, it has only been studied in two monocot species thus far: rice, which is a cereal crop (Tang et al., 2011) and in a forage crop, ryegrass (Jespersen et al., 2016).

The phylogenetic tree revealed that Pyrus PAO proteins could be subdivided into four subfamilies, namely, Group A, B, C, and D. This division of clades is done on the basis of homologous relationships with members of inter- and intra-species. All the clades were shared by members of every genome used in this study. In pepper, the identified CaPAO also showed homology with the AtPAO members. PAO members from other genomes including *Physcomitrella patens*, *Picea sitchensis*, *Selaginella moellendorffii*, *Nicotiana tobacum*, *Glycine max*, *Populus trichocarpa*, and *Solanum lycopersicum* also showed similar homology pattern in phylogenetic tree (Tang et al., 2011).

By comparing Pyrus *PAO* the evolution of the members of this gene family was examined. It was found that almost all genes evolved through segmental duplication. Most of the Pyrus *PAOs* showed segmental duplication. However, at least one member from every single genome showed tandem duplication. In the promoter regions, *cis*-elements contribute to the stress responsiveness to environmental conditions a plant is exposed to. In Pyrus *PAOs*, *cis*-elements associated with light-related, hormone-related, stress-related, and development-related responsiveness were found which showed their involvement in abiotic as well as biotic stress responsiveness. The similar *cis*-elements have also been observed in *PAO* members from *Arabidopsis* which indicates their functional similarity and conservation across species (Aubry et al., 2018). Further, the functional prediction though PPI and GO analysis also showed the conservation and involvement of these genes in many metabolic reactions that take place in a leaf during senescence and loss of chlorophyll. The 3D structures necessary for performing proper functions was also found to be highly conserved among all members which also confirms the functional conservation among members identified from Pyrus genomes.

Pyrus plants are susceptible to a variety of biotic and abiotic challenges that can impair their growth and development, resulting in the loss of chlorophyll, cell death, and ultimately early senescence. The quality and productivity of crop plants would be impacted by each of these outcomes. Stresses of many kinds can have an impact on the expression and activity of *PAO*, an essential intermediary in the breakdown of chlorophyll. Apple leaves under drought had a significant upregulation in the relative expression of and *PAO* (Wang et al., 2013). According to microarray study, *PAO* overexpression in response to different stressors coincided with the decomposition of chlorophyll under these circumstances (Thomas et al., 2001). RNA-seq expression data analysis revealed that Pyrus DaPAO genes express differently in different tissues, disease condition, and the abiotic stresses (drought). The *DaPAO6* and *DaPAO8* were highly expressed in tissues while *DaPAO1* exhibited down expression. In canola, *BnPaO1* transcripts were only detectable in the early stages of seed development, but *BnPaO2* expression was observable in seeds throughout seed development. When comparing *BnPaO2* transcripts to *BnPaO1* transcripts, the former displayed expression levels around 5.5 times greater, while the latter displayed levels of expression comparable to 21 DAP canola seeds at 8 to 10 DAP (Ho et al., 2006). Similarly, *DaPAO4* and *DaPAO5* showed an upregulation in normal and diseased fruit conditions. Moreover, in drought stress, four genes *DaPAO2*, *DaPAO3, DaPAO4*, and *DaPAO5* showed an upregulation in gene expression while *DaPAO1* showed a downregulation. Thus, these genes expressed significantly in abiotic and biotic stress conditions as well as in different developmental stages. Therefore, Pyrus *PAO* genes can be used in further research as this study has revealed their important role in stress responsiveness. In order to improve agricultural stress resilience, breeding and genetic engineering efforts involving the selection and integration of these genes may benefit greatly from the insights provided by these structural and functional perspectives.

## 5 Conclusion

PAO is a crucial enzyme in the chlorophyll catabolism process. It ultimately generates the principal fluorescent chlorophyll catabolite and opens the porphyrin macrocycle. The present study provides a systematic as well as comparative analysis of PAO genes in eight economically important and nutritious Pyrus genomes. A total of 11 genes from Cuiguan genome (CuPAO), eight from Shanxi Duli (ShPAO), ten from Zhongai1 (ZhPAO), ten from Nijisseiki (NiPAO), ten from Yunhong No.1 (YuPAO), nine from d’Anjou (AnPAO), five from Bartlett v2.0 (BrPAO), and eight from Dangshansuli’ v.1.1 genome (DaPAO) were identified. We examined the physicochemical characteristics, evolutionary relationships, structural and functional conservation of these Pyrus PAO gene family members. Further, the cis-regulatory elements and expression analysis were also performed to analyze their expression in different stresses. These results are helpful to understand the roles of PAO genes in the various tissues, disease condition, and abiotic stress (drought). *DaPAO5*, *DaPAO6*, *DaPAO7*, and *DaPAO8* are potential candidates which could help the pear confer tolerance against disease as well as drought stress. These genes can be subjected to genetic engineering research to create drought- and disease-tolerant crops, but more research is required to fully understand them. Additionally, our research will aid in the ongoing research on Pyrus’s PAOs functional roles to develop stress resistant Pyrus varieties.

## Data Availability

The original contributions presented in the study are included in the article/Supplementary Material, further inquiries can be directed to the corresponding authors.
